# Experimental evidence of gradual size‐dependent shifts in body size and growth of fish in response to warming

**DOI:** 10.1111/gcb.14637

**Published:** 2019-04-29

**Authors:** Magnus Huss, Max Lindmark, Philip Jacobson, Renee M. van Dorst, Anna Gårdmark

**Affiliations:** ^1^ Department of Aquatic Resources Swedish University of Agricultural Sciences Öregrund Sweden

**Keywords:** Baltic Sea, body size, climate change, coastal ecosystem, fish, life history, population, temperature, temperature–size rule

## Abstract

A challenge facing ecologists trying to predict responses to climate change is the few recent analogous conditions to use for comparison. For example, negative relationships between ectotherm body size and temperature are common both across natural thermal gradients and in small‐scale experiments. However, it is unknown if short‐term body size responses are representative of long‐term responses. Moreover, to understand population responses to warming, we must recognize that individual responses to temperature may vary over ontogeny. To enable predictions of how climate warming may affect natural populations, we therefore ask how body size and growth may shift in response to increased temperature over life history, and whether short‐ and long‐term growth responses differ. We addressed these questions using a unique setup with multidecadal artificial heating of an enclosed coastal bay in the Baltic Sea and an adjacent reference area (both with unexploited populations), using before‐after control‐impact paired time‐series analyses. We assembled individual growth trajectories of ~13,000 unique individuals of Eurasian perch and found that body growth increased substantially after warming, but the extent depended on body size: Only among small‐bodied perch did growth increase with temperature. Moreover, the strength of this response gradually increased over the 24 year warming period. Our study offers a unique example of how warming can affect fish populations over multiple generations, resulting in gradual changes in body growth, varying as organisms develop. Although increased juvenile growth rates are in line with predictions of the temperature–size rule, the fact that a larger body size at age was maintained over life history contrasts to that same rule. Because the artificially heated area is a contemporary system mimicking a warmer sea, our findings can aid predictions of fish responses to further warming, taking into account that growth responses may vary both over an individual's life history and over time.

## Graphical abstract

Using a setup with multidecadal artificial heating of an enclosed coastal bay in the Baltic Sea and an adjacent reference area, our study offers a unique example of how warming can affect fish populations over multiple generations. We found strong but gradual changes in body growth, varying with body size. Because the artificially heated area is a contemporary system mimicking a warmer sea, our findings can aid predictions of fish responses to further warming, taking into account that growth responses may vary both over an individual's life history and over time.

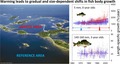

## 1 INTRODUCTION

There is a growing awareness that marine ecosystems and the services they provide are threatened by anthropogenic global climate change (Doney et al., 2012; IPCC, 2014, 2018). Global warming affects many aspects of natural environments, ranging from individual physiology to shifts in species ranges and seasonal life history transitions, and may also bring about evolutionary responses (Daufresne, Lengfellner, & Sommer, 2009; Parmesan & Yohe, 2003; Sheridan & Bickford, 2011). Evidence is accumulating that one such common response to global warming is faster juvenile growth and/or developmental rates and smaller adult body sizes, referred to as the temperature–size rule (TSR) (Atkinson, 1994; Baudron, Needle, Rijnsdorp, & Marshall, 2014; Horne, Hirst, & Atkinson, 2017; Kingsolver & Huey, 2008; Ohlberger, 2013; Tseng et al., 2018). TSR appears to be especially strong in aquatic environments, one contributing factor likely being a stronger effect of oxygen limitation in aquatic than terrestrial systems (Forster, Hirst, & Atkinson, 2012; Horne, Hirst, & Atkinson, 2015). Observations of declines in adult body size in warming environments are also in agreement with long‐known temperature–size relationships in endotherms based on latitudinal gradients, both between species (belonging to the same taxonomic clade: Bergmann, 1847) and between populations of the same species (James, 1970), with organisms generally being smaller in warmer regions (Torres‐Romero, Morales‐Castilla, & Olalla‐Tárraga, 2016; but see Riemer, Guralnick, & White, 2018). In addition to direct physiological effects of temperature on body growth also biotic factors, the most obvious one being food availability, is tightly linked to body growth (Persson & de Roos, 2006). Warming‐induced changes in food availability may thus contribute to warming‐induced changes in fish body growth, also because the optimum temperature for growth increases with food density (Elliot & Hurley, 2001).

There is an increasing number of studies on natural populations finding evidence for relationships between temperature, body growth, and body size in line with the TSR using time‐series analyses (e.g., Baudron et al., 2014; Thresher, Koslow, Morison, & Smith, 2007). However, as most such studies are based on data from commercially harvested species, it is difficult to disentangle the relative contribution of exploitation and temperature, limiting our ability to predict long‐term responses to warming. Similar relationships between temperature, body growth, and size are also found across thermal gradients (e.g., space for time approaches: Blois, Williams, Fitzpatrick, Jackson, & Ferrier, 2013; Meerhoff et al., 2012; van Rijn, Buba, DeLong, Kiflawi, & Belmaker, 2017). Although comparisons across space are valuable and often the only possible empirical approach, this approach suffers from the limiting assumption that the climate variable under study is the only factor that systematically differs among study sites. One rare exception when this assumption may be valid is for natural study systems that vary strongly in the climate variable of interest (e.g., temperature) also within a small area, such as in geothermal ecosystems (O'Gorman et al., 2012). Still, also in the latter approach, we lack the transient link from short‐ to long‐term responses, making analyses of evolutionary adaptations and of gradual changes to warming impossible.

The TSR, predicting a plastic increase in initial body growth but a reduction in adult body size with increasing temperature (Atkinson, 1994), underscores that the nature and strength of climate change effects vary over life history, and that we can only understand population responses to climate change in view of individual development. Still, despite increasing evidence for size‐ and life stage‐specific responses to rising temperatures (Daufresne et al., 2009; Gardner, Peters, Kearney, Joseph, & Heinsohn, 2011; Messmer et al., 2017), most current ecological theory (e.g., Binzer, Guill, Brose, & Rall, 2012; Vasseur & McCann, 2005) aiming to explain population responses to temperature variation is based on the assumption of size‐independent effects of warming (e.g., assuming no temperature dependence of allometric exponents of vital rates, such as metabolic rates; but see Lindmark, Huss, Ohlberger, & Gårdmark, 2018; Lindmark, Ohlberger, Huss, & Gårdmark, 2019; Ohlberger, Edeline, Vollestad, Stenseth, & Claessen, 2011). This may be a serious limitation given that life stage and body size have major influences on the physiology (e.g., Brown, Gillooly, Allen, Savage, & West, 2004) and ecological role of individuals (e.g., Brose, 2010). The ecological role is, in turn, inherently linked to ecological dynamics (de Roos & Persson, 2013).

Here, we analyze long‐term responses in fish body growth and size to warming, and how they vary over ontogeny, using an artificially heated, nonexploited, enclosed ecosystem in the Baltic Sea archipelago exposed to artificial warming during >40 years to date. In a paired design, through annual survey fishing in this heated area and an adjacent reference area (both closed to other types of fishing), we have assembled close to 13,000 individual back‐calculated body growth trajectories of the omnivorous fish species Eurasian perch (*Perca fluviatilis*) caught throughout the period 1969–2004, derived from annual growth rings in their bony structures. This provides a unique opportunity to test whether short‐term growth responses of organisms hold in the long term, as well as whether responses vary depending on individual body size or life stage by using actual growth trajectories of individuals. We report results on size‐dependent growth responses in the Baltic Sea perch population exposed to chronic warming and test the prediction that body growth of small individuals responds stronger and more positively to warming than body growth of large individuals. Also, we test whether shifts in body growth trajectories and size‐at‐age following warming are due to immediate plastic responses or are caused by more gradual changes, for example, through adaption to warmer waters or warming‐induced shifts in the biotic environment. This study offers a unique example of how warming can affect individual body growth and size‐at‐age over multiple generations in an artificially heated enclosed coastal ecosystem relative to an adjacent reference area.

## 2 MATERIALS AND METHODS

### 2.1 Study system

**Figure 1:**
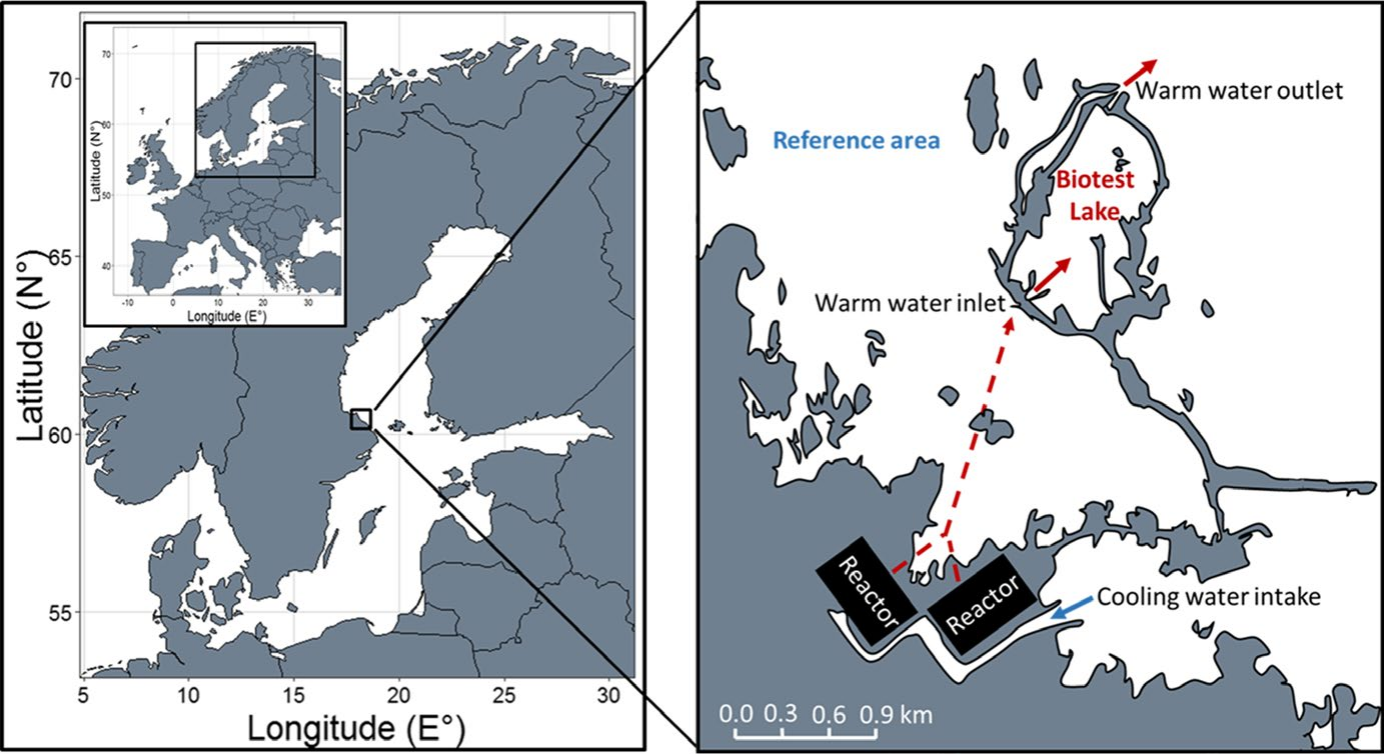
Study area. The location (left) and map (right) of the artificially heated enclosed coastal ecosystem, the Biotest Lake (heated from 1980 onward), and its reference area. Full arrows indicate the warm water inlet and outlet (transported in tubes indicated by hatched arrow)

**Figure 2:**
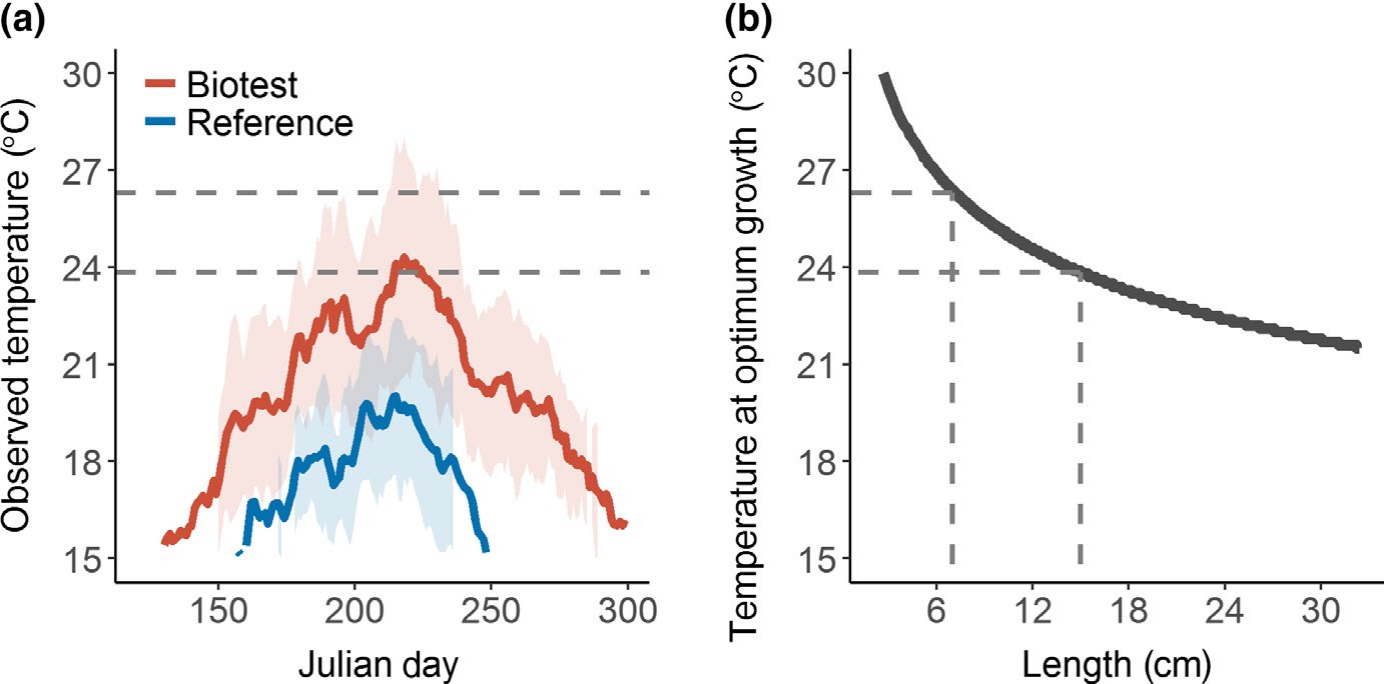
Optimum temperature for growth and daily water temperature. (a) The observed mean (±1*SD*, shaded areas) daily water temperatures in the heated Biotest Lake (red) and its reference area (blue) during the ice‐free season during the time period 1989–2003. (b) The optimum temperature for perch net energy gain (energy available for body growth, see Figure S1) as a function of body length with the mean length and optimum temperatures of 1 and 3 year old perch indicated with dashed lines (70 and 150 mm, respectively)

To study the effects of warming on fish body growth, we used an artificially heated 1 km^2^ enclosed coastal ecosystem in the Baltic Sea archipelago called the Biotest Lake (Figure 1). This enclosure was completed in 1977 and built to receive the heated cooling water from the nearby nuclear power plant in Forsmark, Sweden. Since 1980, when the first reactor was started, the water temperature in the Biotest Lake has been ~5°C to 10°C above that of the surrounding sea (see Figure 2b for an example of the daily difference in temperature over a growth season). The fish communities in the Biotest Lake and in the adjacent reference area (established as a paired design, Figure 1) have been monitored since the construction of the enclosure. There was no significant difference in the rate of increase in temperature over the study period between the Biotest Lake and the reference area during the main growth season (yearly means based on daily measurements for the period May–October in years with temperature records from both areas, linear regression: *F*_1,11_ = 0.93, *p* = 0.36, *r* ^2^ = 0.078). Consequently, we can assume that observed responses to artificial warming are not due to any difference in gradual temperature shifts between the areas.

The fish community in the Biotest Lake is dominated by stationary species with low migration potential and limited home range, the most abundant ones being common roach (*Rutilus rutilus*) and perch (Adill, Mo, Sevastik, Olsson, & Bergström, 2013; Sandblom et al., 2016). Aside from regular survey fishing with stationary gears, no fishing has been allowed in the Biotest Lake or in the reference area since construction was initiated. During the whole study period, the Biotest Lake was closed for fish migration (at least for fish >10 cm, such as adult perch) from the surrounding sea by a grid (removed in 2004) at the outlet, as well as by a strong current (80 to ca 100 m^3^ water/s; Adill et al., 2013) through the grid that prevents immigration of small fish to the Biotest Lake. Combined with studies providing evidence for genetic and physiological differentiation (Björklund, Aho, & Behrmann‐Godel, 2015; Sandblom et al., 2016), this suggests that the two perch populations studied in this paper, inside and outside the heated enclosure, were separated during the study period and have been so for many generations. As found in other comparative studies (e.g., Neuheimer & Grønkjær, 2012), fish size‐at‐age (here of perch) has been shown to be higher in the warmer of the two areas (Adill et al., 2013; Sandblom et al., 2016). Here, we, for the first time, address the long‐term warming effects on actual body growth of perch throughout their life history in the two areas. The unique experimental setup, including multiple years before and after artificial warming started, allowed for a paired before‐after treatment analysis.

Our study species, perch (*P. fluviatilis*), is a common and often numerically dominant fish species in many European lakes and in the brackish coastal waters of the Baltic Sea (Helcom, 2018; Thorpe, 1977). Perch is an omnivorous species that starts out feeding on zooplankton but with increasing size also feeds on macroinvertebrates and finally becomes piscivorous (Persson, 1988). The maximum reported age of perch is 22 years (http://www.fishbase.org), but in the Biotest Lake, the average life expectancy was found to be 10.8 years prior to artificial warming and 9.6 years after warming (Sandström, Neuman, & Thoresson, 1995). As for many fish species, there is considerable variation in perch life history traits among populations, for example, depending on density‐dependent processes and food availability (Byström, Persson, & Wahlström, 1998), latitude (Heibo, Magnhagen, & Vøllestad, 2005), and as in our case, temperature. Maturation age of perch ranges from 2 to 5 years (Heibo & Magnhagen, 2005; Sandström et al., 1995). For small perch, the optimum temperature for growth is close to 30°C in laboratory environments, but the optimum decreases with increasing body size (Karås & Thoresson, 1992; Figure 2).

### 2.2 Fish sampling

We assembled fish data that has been continuously collected since the 1970s. These include catch per unit effort per species (CPUE, number of fish per net per night), length‐at‐age of perch when caught, and back‐calculated individual somatic growth of perch derived from measured annuli in their operculum bone. Fish sampling in the Biotest Lake was conducted during 3 years prior to warming (1977–1979) and continuously so after warming started in 1980. In the reference area, fish sampling started already in 1970. The sampling and aging methods used are described below.

#### 2.2.1 Catch per unit effort

Multimesh survey gillnets were used to estimate survey catch per unit effort for the period 1977–2004 in the Biotest Lake and the reference area (Söderberg, 2009; Thoresson, 1996). These consist of two 35 m long and 3 m deep linked stationary nets, each with five different mesh sizes (17, 22, 25, 33, and 50 mm). Net locations (five in the Biotest Lake and four in the reference area) were randomly selected within different depth strata. In the Biotest Lake, we only used three of these for CPUE estimates based on their continuity in the time series. Fishing occurred overnight and was conducted during 2 weeks (six nights in total), mainly in October when temperatures are relatively stable and the power plant runs with few disturbances (Söderberg, 2009). We used only data from the first fishing night in October to calculate CPUE, in order to avoid any bias in our estimates of fish community composition due to species‐specific declines in catch rates over the 2 week fishing period. For the reference area, we used CPUE data sampled using the same gear as in the Biotest Lake, but caught in August, again only using data from the first nights sampling for each month (see Olsson, Bergström, & Gårdmark, 2012, for details on survey fishing in the reference area). August sampling in the reference area was used as it is most comparable to the Biotest Lake community in October due to similar temperatures (Figure 2b), as temperature is known to affect the activity of fish and therefore catchability of passive gears. We only included species that were continuously sampled in both areas and omitted those that are not representatively sampled by the gillnets (due to behavior or body shape, e.g., pike, *Esox lucius*, and eel, *Anguilla anguilla*) from the analyses. We also excluded any sampling where total catchability could have been impacted due to, for example, too much vegetation or storms.

#### 2.2.2 Age and back‐calculated growth

Fish used to back‐calculate size‐at‐age throughout each individual's growth history were sampled with survey gillnets and fyke nets (all stationary gears) from 1977 and 1970 onward in the Biotest Lake and reference area, respectively (Thoresson, 1996). However, as we used back‐calculated individual growth data from the operculum bone of these fish (starting at year of birth), we have data on body growth of perch caught in the Biotest Lake that were born as early as in 1969 (only two perch individuals born in 1968, hence that year was excluded from all analyses). This resulted in 12 years of growth data before and 24 years of growth data after the onset of warming. In the reference area, the first birth year of perch was 1962, but for analyses including a comparison to perch from the Biotest Lake we only used individuals born during the period 1969–2003. The individuals in all survey catches were sorted by sex. Females were sampled for age determination and growth measurements of operculum annuli throughout the study period but males only for part of it. Thus, for analyses of size‐at‐age and individual body growth, we only include females. This also ensures that any shift in mean size‐at‐age due to warming is not due to a shift in sex ratio (perch is sexually size dimorphic, Heibo & Magnhagen, 2005).

Winter year rings in the operculum bone were used to back‐calculate size‐at‐age for each sampled individual. This allowed reconstruction of each individual's growth trajectory throughout life until being caught (Thoresson, 1996). Back‐calculated length‐at‐age was derived based on the body proportional hypothesis (in our case, a nonlinear version with a power function, Thoresson, 1996), assuming that the ratio of body size to an expected body size given the scale size is maintained throughout life (Francis, 1990; Tarkan, Gaygusuz, Acipinar, & Gursoy, 2006; Thoresson, 1996). The back‐calculation included assessing length–weight and age–length relationships to check for errors. For a smaller subset of the individuals for which growth measurements were taken, age was in parallel determined and validated using winter year rings in otoliths (Thoresson, 1996). We calculated individual yearly length‐specific body growth (*G*_*L*_) as: $G_{L}=\frac{L_{t+1}-L_{t}}{L_{t}}$. We did not assess growth during the survey year, as such estimates would depend on the time of sampling within the year, which was not the same across all years. This resulted in 39,035 length‐at‐age estimates, rendering growth histories of 8,584 unique individuals in the reference area, and 13,400 length‐at‐age estimates and 4,202 unique individual growth histories in the Biotest Lake. In 1970–1991, fish were sampled for age and growth analyses to reflect the size distribution in the catch (Thoresson, 1996). After 1991, a fixed number of fish per size class were selected for age and growth calculations (Andersson, 2015). Such a subsampling can affect estimates of length‐at‐age obtained from length and age when caught (Bettoli & Miranda, 2001), although stratification does not lead to a bias generally and still can provide meaningful comparisons of growth (Nate & Bremigan, 2005). Length stratification may have an effect also on back‐calculated length‐at‐age from individual growth trajectories measured on annuli in bony structures, but in contrast to for length‐at‐age in catch data there are no established correction methods for back‐calculated length‐at‐ages. Also, because the same subsampling strategy was used in both the heated and the reference area, it has no bearing on our conclusions on the difference in growth (estimated from back‐calculated length‐at‐ages) resulting from the difference in temperature.

Because growth is size dependent, we selected one set of back‐calculated growth trajectories within a defined size class for each age, to compare how body growth responses to warming varied over ontogeny. Individuals were grouped into discrete and nonoverlapping size classes (i.e., all individuals within each size class are of the same age), with one size class for each age to ensure that size‐dependent growth rates were not confounded by growth histories of different length. We selected the limits for the size classes within each age to obtain large numbers of individuals for all studied size classes and ages: 0 year olds (5 mm, *N* = 5–329 for each year and area), 1 year olds (65–75 mm, *N* = 4–94), 2 year olds (110–130 mm, *N* = 4–91), and 3 year olds (140–160 mm, *N* = 4–72). In older age classes, there were no nonoverlapping size classes resulting in *N* > 3 for all study years, which is why these age classes were not analyzed for site‐specific differences in growth rates (but see Figure S3e, for length‐specific growth rates of all 190–210 mm 4 year olds). The use of discrete size classes with a high number of individuals of similar size from both areas throughout the study period may also reduce the influence of temporal shifts in length distributions on body growth estimates. For estimates of first‐year growth rates, we assumed size‐at‐hatching to be 5 mm (Huss, Persson, & Byström, 2007). These results are reported as size‐specific growth rates grouped by size class and age to allow for comparison of small (young) versus large (old) perch individuals.

### 2.3 Statistical analyses

The effects of artificial warming on perch size‐at‐age and size‐specific body growth were evaluated by comparing post‐ to pre‐manipulation trends in the Biotest Lake while controlling for concurrent changes occurring in the reference area that was never exposed to artificial warming (i.e., a before‐after control‐impact paired series [BACIPS] design; Thiault, Kernaléguen, Osenberg, & Claudet, 2017). The before‐after warming comparison allows us to determine how perch body size and growth changed from its historical condition, and the comparison with the reference site allows us to discriminate such changes due to the experimental whole‐ecosystem warming in the heated area from those caused by natural variability and underlying trends (due to, e.g., climate change or eutrophication) in common for the whole coastal area (see Olsson et al., 2012 for an analysis of large‐scale environmental covariates of long‐term trends in the reference area and other natural coastal sites). To specifically enable us to discriminate between different time‐dependent effects (i.e., a sudden or gradual growth response) following warming, we applied a Progressive‐Change BACIPS (Thiault et al., 2017). Using this approach, we fitted different models (linear, asymptotic, step, and sigmoid, see Table S1) to our dataset, assuming the model with the highest corrected Akaike information criterion (AIC_c_) weight (ω) to be the best (most parsimonious) model (Burnham & Anderson, 2002). Note that the step‐change model is equivalent to the traditional approach of comparing differences before and after impact using a *t* test or ANOVA. If the best model is the step‐change one, it indicates that there is only an immediate response to warming and no gradual response, whereas any of the other three models represents different types of gradual responses (Thiault et al., 2017). Progressive‐change BACIPS analyses were performed using packages MINPACK.LM (version 1.2‐1, Elzhov, Mullen, Spiess, & Bolker, 2013), NLS.2 (Grothendieck, 2013), and AIC_CMODAVG_ (Mazerolle, 2016) in R version 3.4.3 (R Core Team,^2017^).

Because density‐dependent processes and interactions with other species can affect body growth, we also addressed potential shifts in both perch CPUE (Figure S4) and fish community composition (Figure S5), as an additional (indirect) explanation for divergence in body size and growth between areas following warming (due to lack of prey data over time, we could not address food availability). To get a single measure of the species abundance‐based composition of the fish communities in the two areas, we used principal coordinates analysis (PCoA, Zuur, Ieno, & Smith, 2007) For these to be comparable between the two areas, we applied a single PCoA to species‐specific CPUE from both areas, for species that occurs in both areas. PCoA were made based on Chord distance, which is the Euclidean distance between normalized site (here year) vectors (Legendre & Legendre, 1998) and thus is a metric dissimilarity index that can handle that the total abundances of species vary in time. Using Chord distance also avoids identifying similarities between years due to species absence in multiple years. The first PCO axis (PCO1) explained 57% and the second (PCO2) 33%, with scores representing variation in species composition over time and between areas. For each of the PCO axes, we extracted the site (year) scores for each area to obtain annual measures of species composition on a scale that is comparable between the two areas. Thereafter, we applied Progressive‐Change BACIPS analyses on (a) the difference in perch CPUE between the two areas, and (b) the difference in PCO1 values as well as in PCO2 values, to identify time‐dependent effects following warming.

## 3 RESULTS

**Figure 3:**
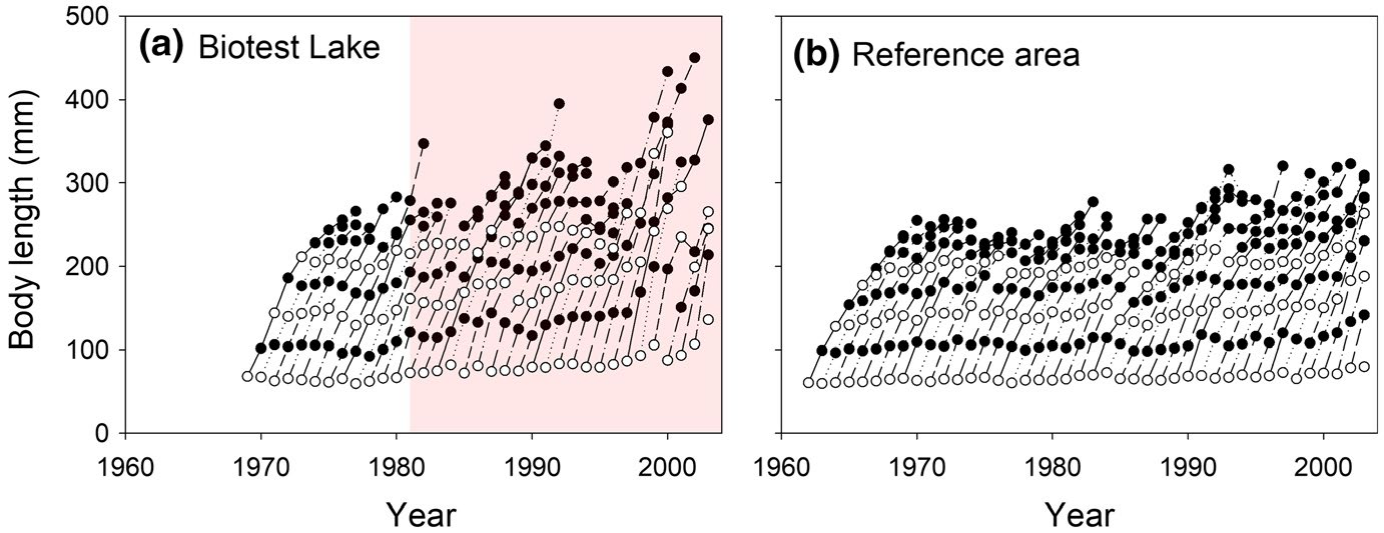
Warming effects on fish growth trajectories. Growth trajectories of different cohorts (each point representing mean length‐at‐age from back‐calculated individual growth trajectories and each line one cohort) of perch in (a) the artificially heated enclosed coastal ecosystem, the Biotest Lake (1969–2004), and (b) its adjacent reference area (1962–2004). The first point in each (cohort) line represent 1 year old individuals, the second 2 year olds, etc. 1, 3 and 5 year old individuals are highlighted in white. The light red area indicates the period during which the Biotest Lake received warm water

**Figure 4:**
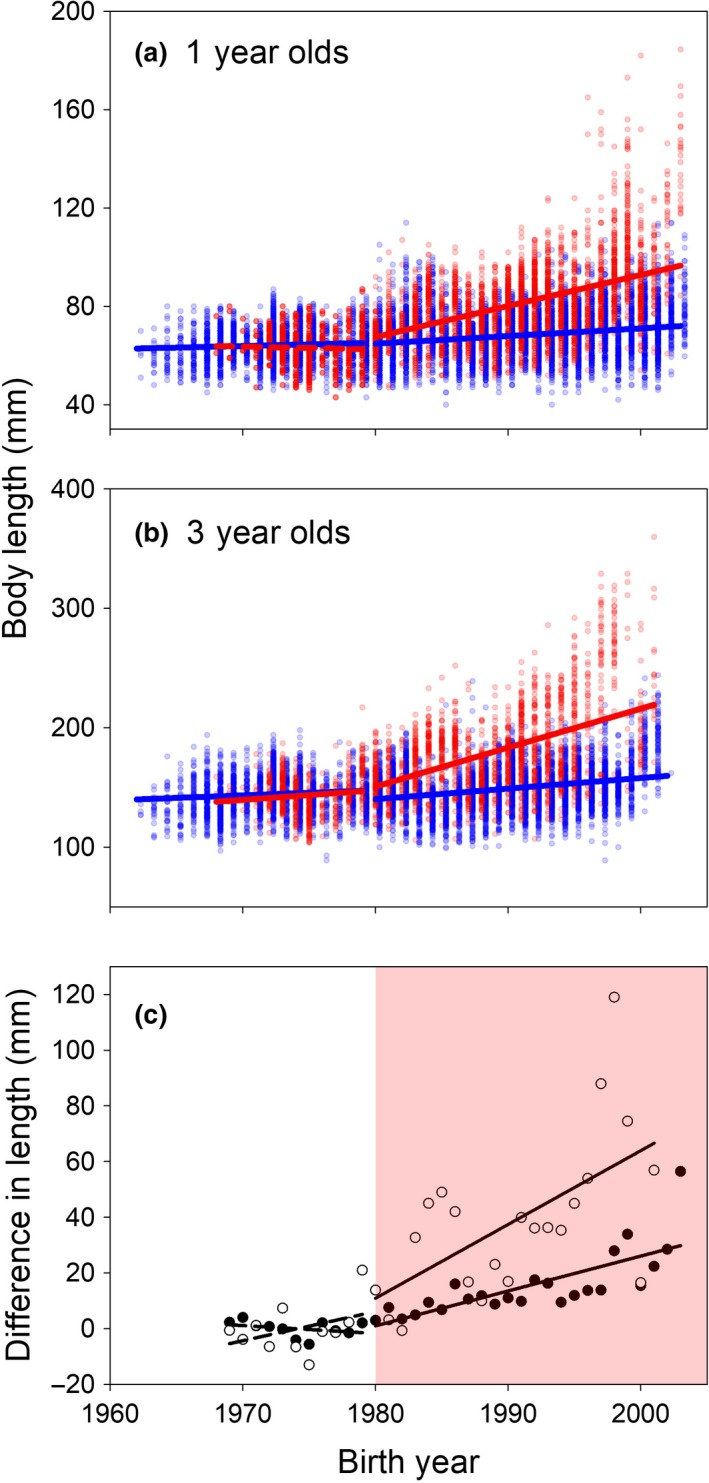
Warming effects on fish size‐at‐age. Body lengths, based on back‐calculated length‐at‐age, of (a) 1 year old perch and (b) 3 year old perch in the artificially heated enclosed coastal ecosystem, the Biotest Lake (red symbols), and its reference area (blue symbols), and (c) the resulting difference in mean body length between areas for 1 year old perch (black symbols) and 3 year old perch (white symbols). Solid regression lines represent significant (*p* < 0.05) relationships. The light red area in (c) indicates the period during which the Biotest Lake received warm water

**Figure 5:**
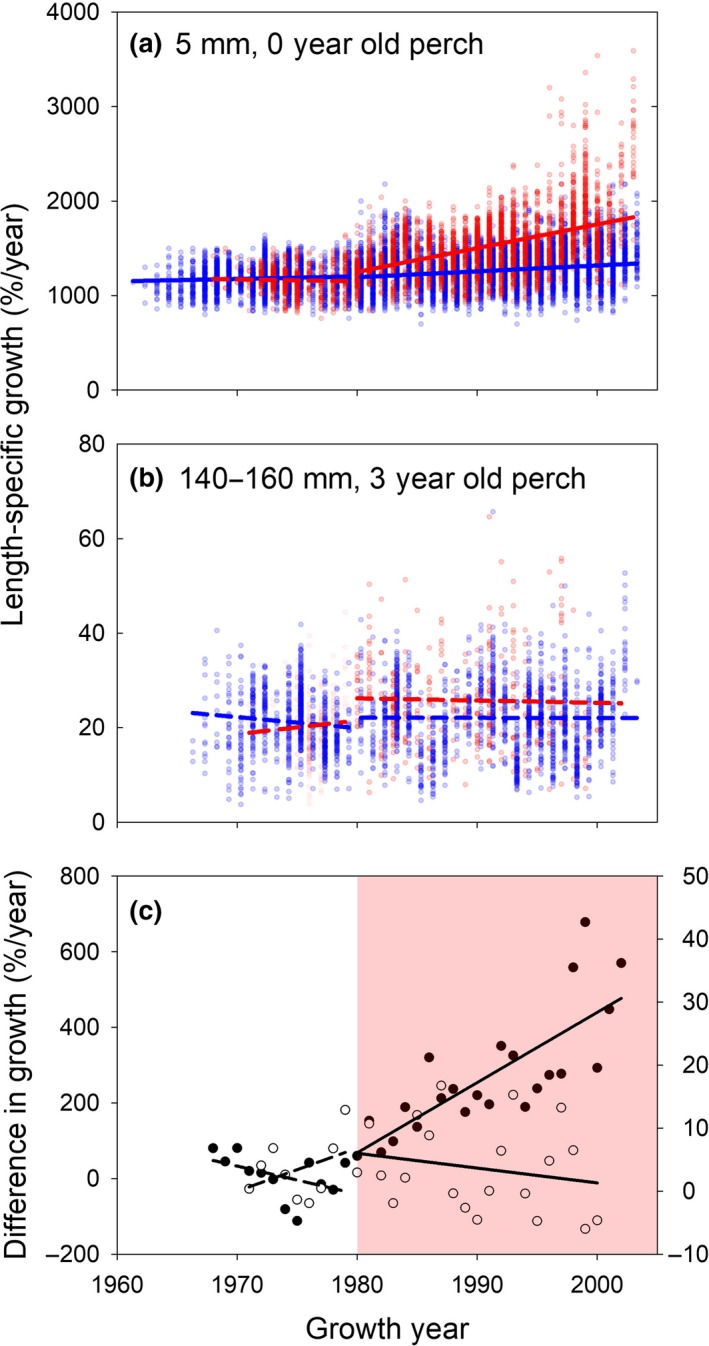
Warming effects on length‐specific body growth of fish. Length‐specific annual growth rates (*G*_*L*_, based on back‐calculated length‐at‐age) of (a) newborn and (b) 140–160 mm 3 year old perch in the artificially heated enclosed coastal ecosystem, the Biotest Lake (red symbols), and its reference area (blue symbols), and (c) the resulting mean difference in body growth between areas for newborn perch (black symbols, left *y*‐axis) and 3 year old perch (white symbols, right *y*‐axis). Solid regression lines represent significant (*p* < 0.05) relationships. The light red area in (c) indicates the period during which the Biotest Lake received warm water

Perch size‐at‐age in the Biotest Lake increased after the onset of warming for all ages studied (1–6 years, but note that we only present responses of 1 and 3 year old fish in the main text), both relative to the prewarming period in the Biotest Lake and relative to the postwarming period in the reference area (Figures 3 and 4, Figure S2). After 24 years of warming, both 1 and 3 year‐old perch were, on average, approximately 35% larger than in the reference area (Figure 4). The Progressive‐Change BACIPS model best supported by data suggests that warming led to a gradual increase in perch body length in the Biotest Lake relative to perch in the reference area, both for 1 and 3 year old individuals (Table 1, Figure 4c). Before artificial heating, there was no significant difference in growth or body size at age between the two areas (i.e., using linear regression analyses we confirmed that the assumption of stationarity before impact was met, see Figures 4c and 5c).

**Table 1:** Best models of time‐dependent effects of warming on size and body growth of fish individuals using step‐change, linear, asymptotic, and sigmoidal models (see Table S1). The best model was selected using corrected Akaike information criterion (AIC_c_) weights (*w*_*i*_) as a measure of the relative likelihood of different models (for *w*_*i*_ for all models, see Table S2)

Response variable	Best model	*w*_*i*_	*R* ^2^	*p*
Size‐at‐age 1	Linear	59.7%	0.71	<0.01
Size‐at‐age 3	Linear	77.1%	0.59	<0.01
Body growth, first year, 5 mm	Linear	60.7%	0.71	<0.01
Body growth, 3 year olds, 140–160 mm	Linear	43.2%	0.015	0.52

Size‐specific growth rate has increased over time for small perch in both areas (Figure 5a,b). However, BACIPS analyses showed that after the onset of warming the size‐specific growth rate of perch increased more in the Biotest Lake relative to in the reference area, but only for younger size/age groups (Table 1, Figure 5, Figure S3). The best models suggest that warming led to a gradual change in body growth both for newborn (i.e., first growth year) perch and for 140–160 mm 3 year old perch (Table 1, Figure 5c). However, there was only a significant gradual increase for 1 year olds and not for 3 year olds.

We found no shift in perch CPUE in the Biotest Lake relative to the reference area (Figure S4). As for the fish community as a whole, there was either a sigmoidal (PCO1, Figure S5) or no (PCO2, Figure S5) shift in fish community composition in the Biotest Lake relative to in the reference area, adding little explanation to the strong linear shifts observed for perch size‐at‐age and size‐specific growth rate of the youngest perch following warming.

## 4 DISCUSSION

Relationships between ectotherm body size and temperature over natural thermal gradients are well documented, but much less is known about how site‐specific warming affects body growth patterns in natural populations. Here, we provide a unique study of wild, unexploited, fish exposed to warming across several generations, by analyzing individual growth patterns of close to 13,000 individuals across 35 years, subject to a large‐scale warming experiment in a paired design with a heated enclosed coastal ecosystem and a reference area lasting 24 years. We found that warming increased growth and thus size‐at‐age, not only immediately following onset of warming, but also that this response increased gradually across the entire 24 year warming period. Moreover, the effect of warming on body growth was strongly size dependent. Fish of all ages increased in average size‐at‐age, but only young, small‐bodied perch exhibited a significant gradual increase in size‐specific body growth relative to perch in the adjacent reference area. Our results suggest that warming, rather than leading to fast, stepwise, plastic changes in body growth across life stages, instead may result in gradual, and substantial, changes varying as organisms grow and develop.

Our findings highlight the fact that the nature and strength of the future climate change effects may vary over life history. A better understanding of how warming effects vary depending on body size is important given that size‐dependent temperature effects yield fundamentally different predictions on how warming affects the dynamics of animal populations (Lindmark et al., 2018, 2019; Ohlberger et al., 2011). Temperature effects that depend on body size can, for example, lead to warming‐induced shifts in the regulation (Lindmark et al., 2018) and dynamics (Ohlberger et al., 2011) of whole populations and communities (Lindmark et al., 2019), by affecting species interactions. Therefore, it is important to quantify temperature effects on, for example, growth and how they depend on body size, and the intraspecific size variation it results in (Lindmark et al., 2018; Ohlberger et al., 2011), for accurate predictions of population responses to warming.

Here, we show, for a natural unexploited fish population, a strong temperature‐dependent relationship between body size, age, and somatic growth rates. In line with several observations suggesting that the thermal optima for body growth in fish decrease with body size (Imsland, Foss, Folkvord, Stefansson, & Jonassen, 2006; Karås & Thoresson, 1992; Morita, Fukuwaka, Tanimata, & Yamamura, 2010; Pörtner & Farrell, 2008, Figure 2b), we found a stronger positive effect of warming on body growth of small than on large perch, as expected under the TSR (Atkinson, 1994). Whether individuals will exhibit faster growth rates or not as temperature increases is also determined by the difference between that optimum and the amount of warming in relation to the ambient temperature. In our case, the ambient (reference) temperature was lower than optimal temperatures for perch individuals of all sizes (Figure 2), suggesting we should expect positive effects of warming. However, given the magnitude of warming in the Biotest Lake, only small individuals, which were furthest away from their optima (Figure 2a), exhibited a strong positive growth response. Indeed, the average temperature in the Biotest Lake during the warmest month (peak growth season) is so high that it exceeds the thermal growth optimum of fish >20 cm (Figure 2) (a size which the average 3 year old perch in the Biotest Lake had reached already in the 1990s, Figure 4b), making strong positive effects of warming less likely for these large fish. Thus, increased size‐at‐age of perch during the warm period was for the larger and older individuals mainly a consequence of fast body growth when young and small. This is in agreement with Angilletta and Dunham (2003), suggesting that constraints in growth rates only occur later in life due to the decreasing optimum temperatures for growth with body size (Björnsson & Steinarsson, 2002). The lack of declining body sizes of older individuals in our study contrasts to the more commonly reported declines in adult body size in response to warming (as predicted by the TSR), although quite some variation in both direction and rate of change between species and systems has been reported (Gardner et al., 2011; Sheridan & Bickford, 2011). Among previous studies reporting increased size‐at‐age, most concern juvenile individuals (including examples with fish, e.g., Thresher et al., 2007) and several are from high latitude areas, which may suggest that longer growing seasons and/or increased resource levels in response to warming may offset negative effects of warming on body size (Sheridan & Bickford, 2011). Our results add to previous short‐term observations, suggesting that positive body size response to warming of larger individuals is a potential outcome also under scenarios with continuous warming. Still, as predicted by the TSR (Atkinson, 1994), our results show that to understand patterns in size‐at‐age over temperature, it is necessary to account for how warming affects growth patterns over ontogeny.

In contrast to previous laboratory‐scale fish experiments that have shown a fast plastic growth response to temperature (Björnsson & Steinarsson, 2002; Laurel, Copeman, Mara, & Iseri, 2017; Morita et al., 2010), our results on a natural and unexploited fish population provide evidence for a gradual and long‐term response, rather than only a sudden growth response. Warming induced by climate change may have contributed to the extent of the observed faster growth in the Biotest Lake, as also growth in the reference area increased slightly over time. However, the setup with a reference site ensures that this result is not an effect of concurrent climate change, as this affects both systems and the analyses were made on the difference in fish growth in the two areas. Although the composition of the fish communities has changed over these decades (Olsson et al., 2012), which may influence species interactions and therefore body growth, the changes in fish community composition among the species common to both areas did not correspond to the onset of warming or did so in a sigmoidal rather than gradual linear manner (Figure S5). Thus, changes in fish community composition (or perch density, i.e., perch CPUE, Figure S4) cannot explain the observed gradual body size shifts following warming. Still, gradual change in prey availability or other unknown environmental factors exhibiting a delayed response may have contributed to observed body growth responses to warming. Indeed, warming can result in faster fish growth also via increased productivity of their prey, as body growth depends on food availability. Such indirect effects of warming can only be addressed through whole‐ecosystem warming experiments, such as herein. However, as we lack data on prey availability through time, we could not disentangle this effect from direct effects of warming on perch physiology or feeding rates. Although we cannot distinguish the particular mechanism by which the increased temperature has affected perch growth, we can conclude that warming is driving the long‐term and gradual increased growth of perch in the heated compared to the unheated area.

Our size‐at‐age estimates may differ somewhat from the actual size‐at‐age of the fish, for example, due to the use of only one out of several existing methods for deriving back‐calculated length‐at‐age estimates from measurement of growth increments in hard structures, which indeed can result in different estimates (Francis, 1990). Unfortunately, we could not compare results from different methods for raising growth annuli to back‐calculated length‐at‐ages, due to a lack of raw measurements of the opercula for most of the time series. However, in this study, all samples from both areas were always assessed using a single method for any specific year (see Section 2). Thus, none of the conclusions drawn is sensitive to any potential systematic errors due to the use of a specific back‐calculation method.

The potentially far reaching implications of altered body sizes for future fish dynamics, production and fisheries yield (Cheung et al., 2013; van Rijn et al., 2017) depend on the rate and extent of such size shifts, including potential for gradual adaptation. While model predictions and small‐scale experiments can provide important insights, the lack of understanding of underlying mechanisms (e.g., Lefevre, McKenzie, & Nilsson, 2017) and experimental tests over relevant spatial and temporal scales limits our ability to make accurate predictions, as well as to adapt management of natural fish populations facing climate change accordingly. Comparing body growth and size distribution patterns of organisms from sites already exposed to different thermal regimes can of course inform on the scope for body size shifts in face of climate change, but says little about the route toward that end result and organisms' potential to gradually adapt. In contrast to most correlation‐based studies on body growth responses of natural populations to warming, our study is not only on a controlled warming experiment in a paired design with a double control (reference period and reference area), but also using unexploited populations. The latter is important as it allows us to rule out shifts in exploitation rates as an explanatory factor for shifting growth rates over time. Fishing reduces size‐at‐age and growth rates both through direct demographic effects and evolutionary responses in exploited species (Jørgensen et al., 2007; Östman et al., 2014; Vainikka, Mollet, Casini, & Gardmark, 2009), depending on the size selectivity of the fishing‐induced mortality (Gårdmark & Dieckmann, 2006). Thus, although it is important to resolve warming responses also of exploited species, it is difficult to disentangle these from the strong selection imposed by fishing. Our long‐term controlled experiment on natural unexploited fish populations shows that warming alone can result in substantial and continuous increase in individual body growth (of small and young individuals) and size‐at‐age.

Whether the long‐term gradual changes in body size are better explained by gradually changing ecological conditions or local adaptation is difficult to decipher without common garden experiments or genetic data on temperature adaptations. Although there is ample evidence for plastic responses to warming, little is known about evolutionary body size responses to warming (Crozier & Hutchings, 2014; Merilä & Hendry, 2014). Many of the studies that find support for plastic temperature responses in fish are based on short time scales making it hard to observe adaptive responses. However, acclimation through phenotypic plasticity, which should manifest itself within one generation, alone can hardly explain our results. Indeed, the gradual shift in body growth occurred over a period equivalent to >10 generations (maturation at age 2; Sandström et al., 1995). While the relative contribution of genetic change and plasticity is unknown in our case, the consistently high selection pressure (i.e., high temperatures) provides one necessary component for evolutionary responses to have occurred. However, evidence for the latter would require both to establish that the shift in growth rates has a genetic basis and that this shift is adaptive (Merilä & Hendry, 2014).

In conclusion, our study offers a unique example on how warming can affect unexploited fish populations over multiple generations in a natural ecosystem. Using measurements of the life‐long growth history from close to 13,000 unique individuals over 35 years, we found a strong increase in growth (depending on body size) in response to artificial heating. Most importantly, this response was clearly gradual, suggesting that other factors than short‐term plastic growth responses need to be taken into account. Our results imply that accurate predictions on fish body growth, size, and production in a future warmer climate requires acknowledging that growth responses may vary both over an individual's life history and continue over time.
