# Supplementary material for: Experimental evidence of gradual size‐dependent shifts in body size and growth of fish in response to warming
Source: Glob Chang Biol. 2019 Apr 29;25(7):2285–95. doi: 10.1111/gcb.14637 (PMC6850025; doi:10.1111/gcb.14637)
Supplement: Supplementary file 1 [file GCB-25-2285-s001.zip › gcb14637-sup-0001-Supinfo1.docx]

**SUPPPORTING INFORMATION**

**Table S1** Different type of responses of size and body growth of fish to warming and the corresponding equations describing the difference between the artificially heated enclosed coastal ecosystem, the Biotest lake, and its reference area after warming of the Biotest lake started in 1980 (*Δwarm*), as a function of the difference before warming (*Δcold*), the time since start of warming (*t*) and parameters affecting the shape and size of the response (*M* = magnitude, *r* = rate of change, *L* = time at which half of the final effect is achieved, *K* = shape parameter). *ɛ* is the error of the expected difference between areas at a specific time step (*t*) after warming.

| **Model** | **Equation** |
| --- | --- |
| *Step-change* | $\Delta warm=M+\Delta cold+\varepsilon_{t}$ |
| *Linear* | $\Delta warm=rt+\Delta cold+\varepsilon_{t}$ |
| *Asymptotic* | $\Delta warm=\frac{Mt}{L+t}+\Delta cold+\varepsilon_{t}$ |
| *Sigmoid* | $\Delta warm=\frac{{M(\frac{t}{L})}^{K}}{{1+(\frac{t}{L})}^{K}}+\Delta cold+\varepsilon_{t}$ |

**Table S2** AICc weights (*w_i_*) as a measure of the relative likelihood of different models of time dependent effects of warming on size and body growth of fish individuals using step-change, linear, asymptotic and sigmoidal models (see Table S1). Selected models are indicated in bold.

| **Response variable** | **Model** | ***w_i_*** |
| --- | --- | --- |
| *Size-at-age 1* | *Step-change* | <0.01% |
|  | ***Linear*** | **59.7%** |
|  | *Asymptotic* | 16.5% |
|  | *Sigmoid* | 23.8% |
| *Size-at-age 3* | *Step-change* | 0.09% |
|  | ***Linear*** | **77.1%** |
|  | *Asymptotic* | 16.3% |
|  | *Sigmoid* | 6.54% |
| *Body growth, first year, 5 mm* | *Step-change* | <0.01% |
|  | ***Linear*** | **60.7%** |
|  | *Asymptotic* | 16.8% |
|  | *Sigmoid* | 22.5% |
| *Body growth, 3-year olds, 140-160 mm* | *Step-change* | 34.7% |
|  | ***Linear*** | **43.2%** |
|  | *Asymptotic* | <0.01% |
|  | *Sigmoid* | 22.1% |

|  |
| --- |


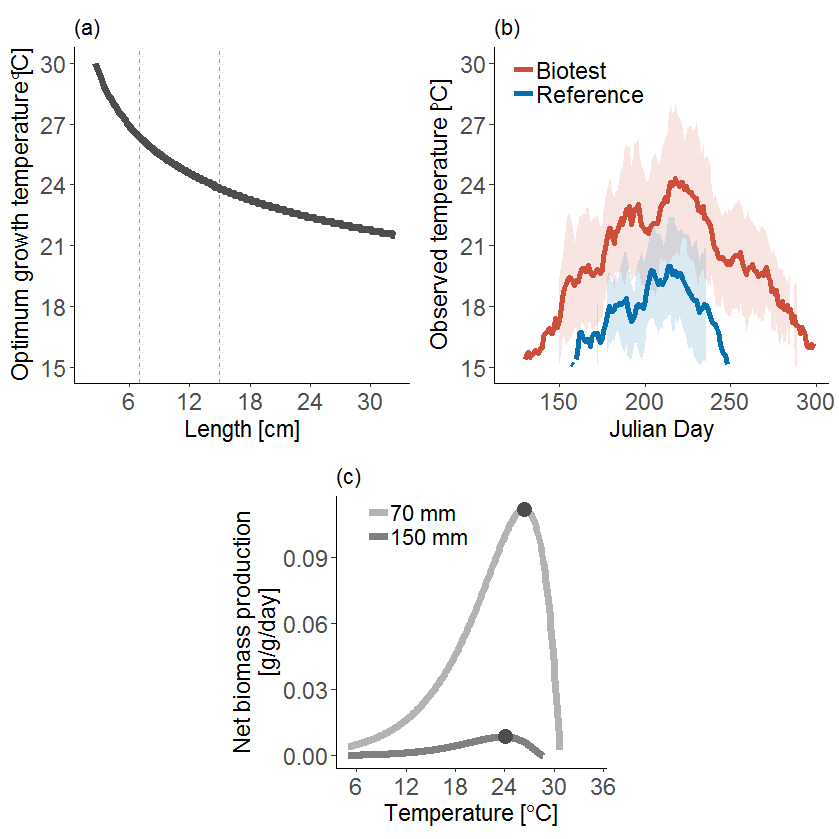


**Figure S1. Optimum temperature for growth and daily water temperature.** Biomass-specific net biomass production as a function of temperature for a 70 mm (light grey) and 150 mm (dark grey) perch, with the optimum temperature for net energy gain indicated by a filled circle.


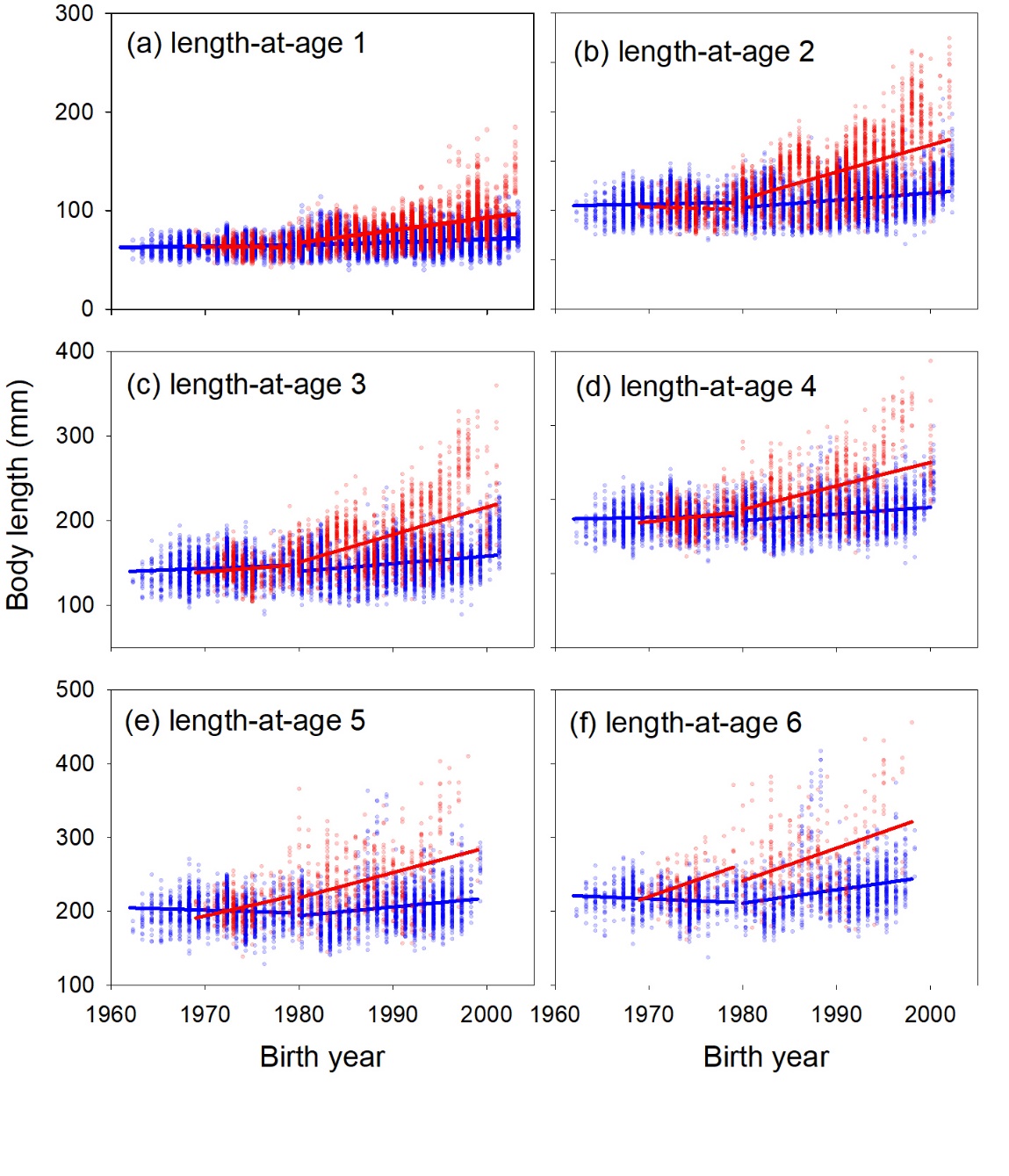


**Figure S2.** **Warming effects on** **fish size-at-age.** Body lengths of one (a), two (b), three (c), four (d), five (e) and six (f) year old perch in the artificially heated enclosed coastal ecosystem, the Biotest Lake (red symbols), and its reference area (blue). Regression lines represent significant (P < 0.05, solid) and non-significant (dashed) linear relationships. Warming in the Biotest Lake started in 1980.


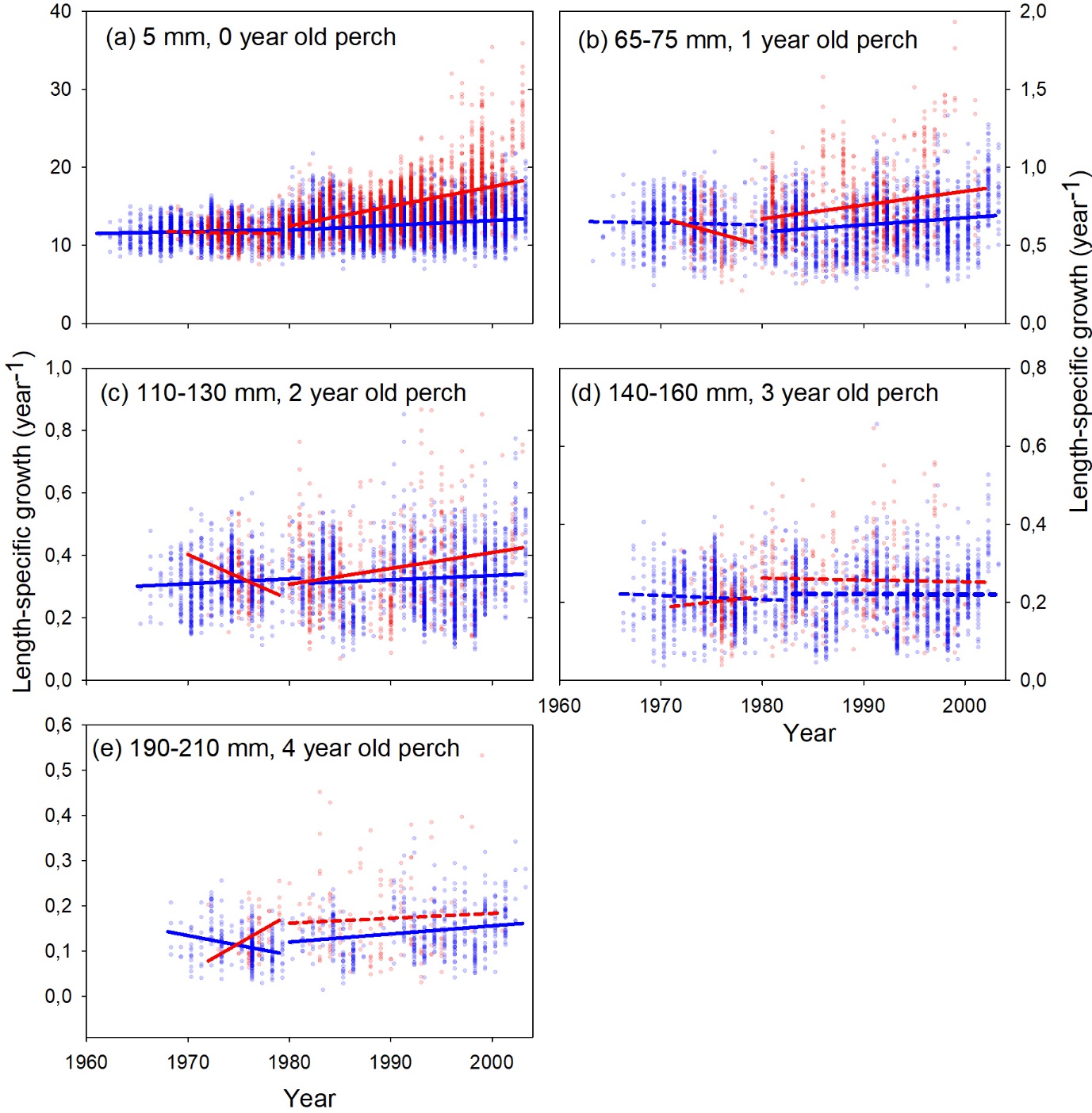


**Figure S3.** **Warming effects on** **length-specific body growth of fish.** Length-specific annual growth rates (*G_L_*) of (a) newborn, (b) 65-75 mm one year old perch, (c) 110-130 mm two year old perch, (d) 140-160 mm three year old perch and (e) 190-210 mm four year old perch in the artificially heated enclosed coastal ecosystem, the Biotest Lake (red symbols), and its reference area (blue symbols). Regression lines represent significant (P < 0.05, solid) and non-significant (dashed) linear relationships. Warming in the Biotest Lake started in 1980.

**Figure S4.** Difference in perch catch per unit effort (CPUE, number of perch net^-1^ night^-1^), between the Biotest Lake and the reference area over time. The difference in CPUE shows no significant trend after warming (best model: linear, *w_i_* = 66.6%, *R^2^* = 0.097, *P* = 0.121). The light red area indicates the period during which the Biotest Lake received warm water.


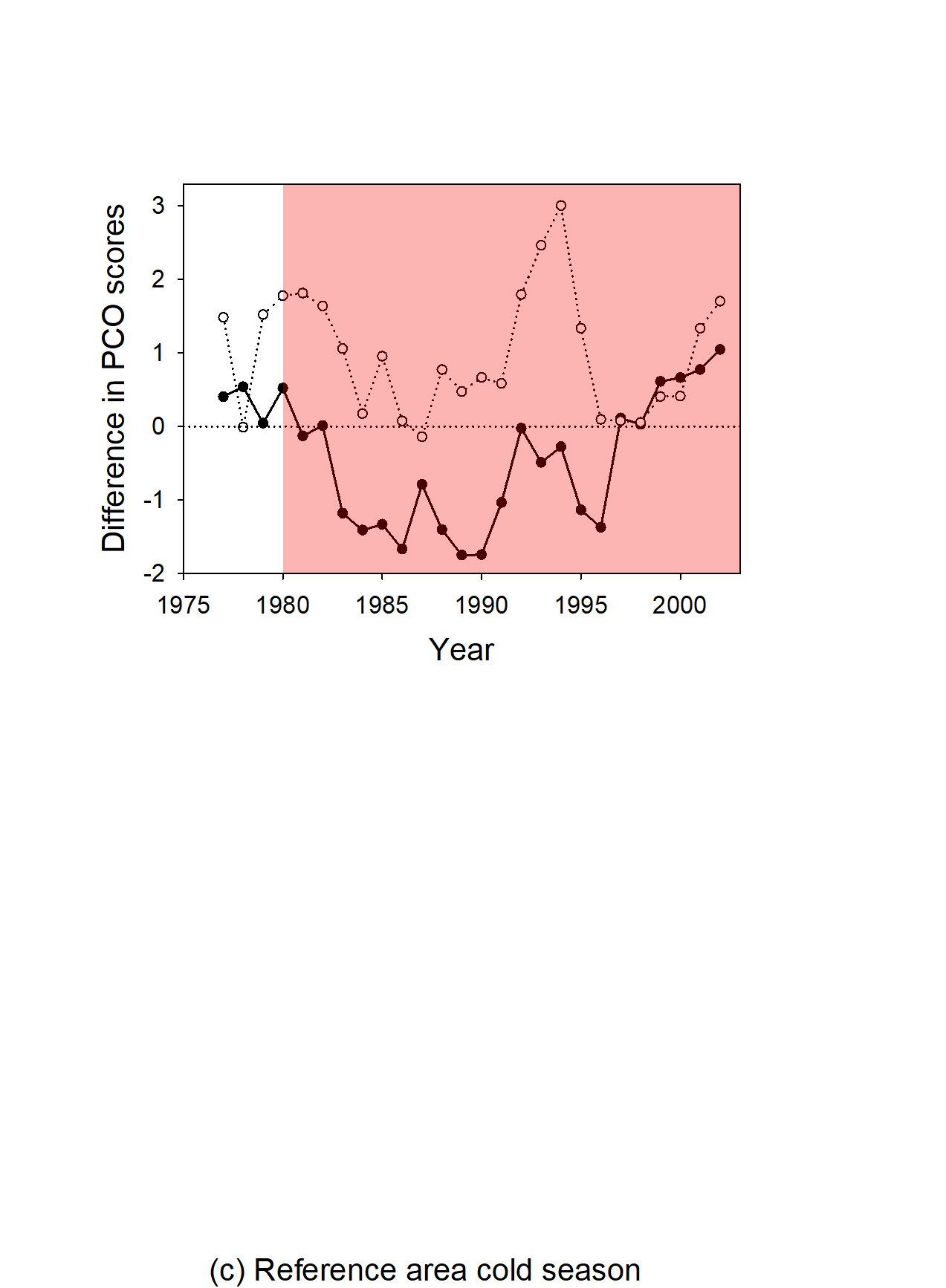


**Figure S5.** Difference in fish species composition between the Biotest Lake and the reference area over time, as described by the difference in PCO scores of the abundance-based species composition among the species in the fish community occurring in both areas. The difference in PCO scores, from neither the first (black) nor the second (white) ordination axes, between the heated and the reference area shows any significant difference or linear trend after warming compared to before onset of warming (best model PCO1: sigmoidal, *w_i_* = 80.9%, best model PCO2: linear, *w_i_* = 48.2%, *R^2^* = 0.004, *P* = 0.751). The light red area indicates the period during which the Biotest Lake received warm water.

**Model of size-dependent optimum growth temperature**

We followed the bioenergetics approach used in Ohlberger et al. (2011) to describe the size ($m$)-and temperature ($T$) dependence of perch body growth rate, via individual-level feeding and metabolic rates. Growth, or per capita net biomass production ($E_{a}(I(m,T)0.5$), is assumed to equal the difference between ingested food $(I(m,T))$ and metabolic costs$(E_{m}(m,T))$, multiplied by a conversion efficiency factor $(0.5)$. The relationship between metabolism and body mass is described with the allometric function$\rho_{1}m^{\rho_{2}}$. Ingestion (i.e. feeding rate) is a function of handling time $(H(m,T))$ and encounter rate $(\omega\left( m,T \right))$ and assumed to follow a Holling type II functional response $(I\left( m,T \right)=\frac{\omega\left( m,T \right)}{1+H(m,T)\omega\left( m,T \right)})$ . Encounter rate is assumed to be the product of size-dependent attack rate $(A_{z})$ [$L day^{-1}$], prey density $\left[ L^{-1} \right]$ and the mass of a single prey item ($3{\cdot10}^{-5}$ g, representing a typical zooplankton). Thus, individual body growth is directly linked to food (zooplankton) availability. However, zooplankton density has no effect on the temperature for optimum growth. The attack rate of perch on zooplankton is modeled as a hump-shaped function of perch body mass (see Table S4 for explanation of parameters used):

$(A_{z}=\hat{A}\left[ \frac{m}{m_{opt}}\exp\left( 1-\frac{m}{m_{opt}} \right) \right]^{\alpha})$

The individual level rates metabolism, handling time and attack rate are all multiplied with size-dependent temperature scaling functions, $r_{m,a}(m, T)$. The latter are derived from Ohlberger et al. (2011), based on the bioenergetics model in Karås & Thoresson (1992), which is equivalent to the model presented in Kitchell et al. (1977) but re-parameterized for Eurasian perch (*Perca fluviatilis*) using data from the coastal region used in this study (Karås & Thoresson, 1992). Note that handling time, in contrast to metabolism and attack rate, decreases with temperature ($\frac{1}{r_{a}(m,T)}$). The functions and parameters describing $r_{m,a}\left( m,T \right)$ are found in Table S3 and S4. See also the R-script (R version 3.4.3) below that implements the model, as defined above, to calculate net energy production and reproduce the relationship between temperature at optimum body growth and perch body size (Fig. S1 and Fig. 2 in the main text).

**Table S3** Functions in the bioenergetics model accounting for the size-dependence of the temperature effects on individual net energy gain, resulting in body growth of perch.

| Term | Expression |
| --- | --- |
| $\boldsymbol{r}\left( \boldsymbol{m,T} \right)^{\boldsymbol{*}}$ | $V\left( m,T \right)^{X(m, T)}e^{X(m,T)(1-V(m,T))}$ |
| $\boldsymbol{V(m,T)}$ | $\frac{T_{max}\left( m \right)-T}{T_{max}\left( m \right)-T_{opt}(m)}$ |
| $\boldsymbol{X(m,T)}$ | $W^{2}\left[ 1+\left( 1+\frac{40}{Y} \right)^{0.5} \right]^{2}{\frac{1}{400}}^{**}$ |
| $\boldsymbol{W}$ | $\left( T_{max}\left( m \right)-T_{opt}\left( m \right) \right)lnQ(m)$ |
| $\boldsymbol{Y}$ | $\left( T_{max}\left( m \right)-T_{opt}\left( m \right)+2 \right)\mathrm{lnQ}$ |
| $\boldsymbol{T}_{\boldsymbol{a,opt}}\boldsymbol{(m)}$ | $\gamma_{a,opt}\left( m \right)^{v_{a,opt}}$ |
| $\boldsymbol{T}_{\boldsymbol{a,max}}\boldsymbol{(m)}$ | $\gamma_{a,max}\left( m \right)^{v_{a,max}}$ |
| $\boldsymbol{Q}_{\boldsymbol{a}}\boldsymbol{(m)}$ | $\vartheta_{a}\left( m \right)^{\theta_{a}}$ |
| $\boldsymbol{T}_{\boldsymbol{m,opt}}\boldsymbol{(m)}$ | $\gamma_{m,opt}\left( m \right)^{v_{m,opt}}$ |
| $\boldsymbol{T}_{\boldsymbol{m,max}}\boldsymbol{(m)}$ | $\gamma_{m,max}\left( m \right)^{v_{m,max}}$ |
| $\boldsymbol{Q}_{\boldsymbol{m}}\boldsymbol{(m)}$ | $\vartheta_{m}\left( m \right)^{\theta_{m}}$ |

* the function $r(m,T)$, scaling rates with mass and temperature, was set to equal 1 at 20$^{\circ}C$ by multiplying with $1.804$ (metabolism) and $1.675$ (feeding functions)

** 40 and 400 where changed to 20 and 200 respectively for intake rate parameters, following Ohlberger et al. (2011) and Karås & Thoresson (1992)

***Table S4*** *Parameter values used in the bioenergetics model and allometric functions*

| Parameter | Value | Unit | Interpretation | Source |
| --- | --- | --- | --- | --- |
| Temperature scaling |  |  |  |  |
| Consumption |  |  |  |  |
| $\boldsymbol{\vartheta}_{\boldsymbol{a}}$ | $2.8$ | $-$ | Allometric scalar of $Q_{a}$ | Karås and Thoresson 1992 |
| $\boldsymbol{\theta}_{\boldsymbol{a}}$ | $0.072$ | $-$ | Allometric exponent of $Q_{a}$ | Karås and Thoresson 1992 |
| $\boldsymbol{\gamma}_{\boldsymbol{a,max}}$ | $32$ | $g^{-v_{a,max}}^{\circ}C$ | Allometric scalar of $T_{max}$ | Karås and Thoresson 1992 |
| $\boldsymbol{v}_{\boldsymbol{a,max}}$ | $-0.029$ | $-$ | Allometric exponent of $T_{max}$ | Karås and Thoresson 1992 |
| $\boldsymbol{\gamma}_{\boldsymbol{a,opt}}$ | $28$ | $g^{-v_{a,opt}}^{\circ}C$ | Allometric scalar of $T_{opt}$ | Karås and Thoresson 1992 |
| $\boldsymbol{v}_{\boldsymbol{a,opt}}$ | $-0.043$ | $-$ | Allometric exponent of $T_{opt}$ | Karås and Thoresson 1992 |
| Metabolism |  |  |  |  |
| $\boldsymbol{\vartheta}_{\boldsymbol{m}}$ | $2$ | $-$ | Allometric scalar of $Q_{m}$ | Karås and Thoresson 1992 |
| $\boldsymbol{\theta}_{\boldsymbol{m}}$ | $0.073$ | $-$ | Allometric exponent of $Q_{m}$ | Karås and Thoresson 1992 |
| $\boldsymbol{\gamma}_{\boldsymbol{m,max}}$ | $36.5$ | $g^{-v_{m,max}}^{\circ}C$ | Allometric scalar of $T_{max}$ | Karås and Thoresson 1992 |
| $\boldsymbol{v}_{\boldsymbol{m,max}}$ | $-0.013$ | $-$ | Allometric exponent of $T_{max}$ | Karås and Thoresson 1992 |
| $\boldsymbol{\gamma}_{\boldsymbol{m,opt}}$ | $32$ | $g^{-v_{m,opt}}^{\circ}C$ | Allometric scalar of $T_{opt}$ | Karås and Thoresson 1992 |
| $\boldsymbol{v}_{\boldsymbol{m,opt}}$ | $-0.029$ | $-$ | Allometric exponent of $T_{opt}$ | Karås and Thoresson 1992 |
| Allometric functions |  |  |  |  |
| $\boldsymbol{\alpha}$ | $0.62$ | $-$ | Allometric exponent of planktivory | Claessen et al. 2000 |
| $\hat{\boldsymbol{A}}$ | $3E4$ | $L \mathrm{day}^{-1}$ | Max. zooplankton attack rate | Claessen et al. 2000 |
| $\boldsymbol{w}_{\boldsymbol{opt}}$ | $8.2$ | $g$ | Optimal forager size | Claessen et al. 2000 |
| $\boldsymbol{\varepsilon}_{\boldsymbol{1}}$ | $5$ | $g^{1-\varepsilon_{2}}\mathrm{da}y^{-1}$ | Allometric scalar of handling | Claessen et al. 2000 |
| $\boldsymbol{\varepsilon}_{\boldsymbol{2}}$ | $-0.8$ | $-$ | Allometric exponent of handling | Claessen et al. 2000 |
| $\boldsymbol{\rho}_{\boldsymbol{1}}$ | $0.033$ | $g^{1-\rho_{2}}\mathrm{da}y^{-1}$ | Allometric scalar of metabolism | Claessen et al. 2000 |
| $\boldsymbol{\rho}_{\boldsymbol{2}}$ | $0.77$ | $-$ | Allometric exponent of metabolism | Claessen et al. 2000 |

**References**

Claessen D, de Roos AM, Persson L (2000) Dwarfs and giants: cannibalism and competition in size-structured populations. *The American Naturalist*, **155**, 219-237.

Karås P, Thoresson G (1992) An application of a bioenergetics model to Eurasian perch (Perca fluviatilis L.). *Journal of Fish Biology*, **41**, 217-230.

Kitchell JF, Stewart DJ, Weininger D (1977) Applications of a bioenergetics model to yellow perch (Perca flavescens) and walleye (Stizostedion vitreum vitreum). *Journal of the Fisheries Board of Canada*, **34**, 1922-1935.

Ohlberger J, Edeline E, Vøllestad LA, Stenseth NC, Claessen D (2011) Temperature-driven regime shifts in the dynamics of size-structured populations. *The American Naturalist*, **177**, 211-223.

**Water temperature in the Biotest Lake and reference area**

The daily average water temperatures in the Biotest Lake and reference area were acquired from temperature loggers that are in place during the ice-free season. We selected one station from each area in order to make the time series as long as possible. Temperature readings from depths outside the interval 1-1.5 m were removed in order to make the areas comparable. Averages temperatures for each Julian day were calculated across years 1989-2003. Due to the selection criteria above, these mean daily temperatures were missing in the reference area for the years 1991, 1992, 1995, 1996 and 1999. See supplementary R-script and data (R version 3.4.3) for calculations of the average temperatures per Julian Day in the abovementioned time period.
